# Putting *family* into family-based obesity prevention: enhancing participant engagement through a novel integrated knowledge translation strategy

**DOI:** 10.1186/s12874-018-0588-5

**Published:** 2018-11-08

**Authors:** Kathryn Walton, Tory Ambrose, Angela Annis, David WL. Ma, Jess Haines, Guelph Family Health Study Family Advisory Council; on behalf of the Guelph Family Health Study

**Affiliations:** 1 Department of Family Relations and Applied Nutrition, University of Guelph, Guelph, ON Canada; 2 Department of Human Health and Nutritional Sciences, University of Guelph, Guelph, ON Canada; 3 University of Guelph, 50 Stone Rd. E, Guelph, ON N1G 2W1 Canada

**Keywords:** Integrated knowledge translation, Childhood obesity, Participatory research, Participant engagement

## Abstract

**Background:**

With 1 in 4 Canadian preschoolers considered overweight or obese, identifying risk factors for excess weight gain and developing effective interventions aimed at promoting healthy weights and related behaviours among young children have become key public health priorities. Despite the need for this research, engaging and maintaining participation is a critical challenge for long-term, family-based studies. The aim of this study is to describe the implementation and evaluation of a parent-only advisory council designed to engage participants in the implementation and evaluation of a longitudinal, family-based obesity prevention intervention.

**Methods:**

A Family Advisory Council (*n* = 14 parents, 70% mothers, 64% white), was established to engage participant stakeholders in decisions related to research protocols and strategies to engage and sustain family participation. Using a mixed methods approach, including a participant survey and focus group, we examined the council members’ perceptions of their role and the impact this novel integrated Knowledge Translation (iKT) strategy had on the Guelph Family Health Study (GFHS), a longitudinal family-based study.

**Results:**

All members of the Family Advisory Council felt the topics discussed were appropriate, felt that their opinions were valued and that their suggestions have had an impact and direct benefit on the GFHS. The addition of the Family Advisory Council led to changes in study protocol (i.e. creation of more detailed intervention emails, creation of kid-friendly accelerometer bands) that may have contributed to the high retention rate of the GFHS (95% at 6-month follow-up).

**Conclusions:**

Engaging parents as research partners in family-based research studies may be an effective way to increase participant engagement and study retention.

## Background

The prevention of excess weight gain in early life has become an important public health focus, due to the high prevalence of childhood obesity [1, 2]. The home environment plays a key role in shaping children’s weight-related behaviours, including their eating, activity and sleep behaviours [3]. Parents are the most knowledgeable about the home environment and the family dynamics that influence their family’s motivations and barriers towards behaviour change [4, 5]. Thus, engaging parents as active agents in the research process is crucial for the success of family-based obesity prevention interventions, especially those with longitudinal designs.

Commonly, obesity interventions that are found to be successful from a services perspective (i.e., feasible) fail to translate into meaningful weight-change outcomes (i.e. decreased body mass index; BMI), often because the intervention is not relevant to or practical for participants [6, 7]. As a result, public health, research and granting agencies have emphasized the importance of integrated Knowledge Translation (iKT). iKT is an approach to research that involves knowledge users as equal partners alongside researchers, with the goal of creating more relevant and useful interventions and better research outcomes [8, 9]. While parents (usually mothers) have often been consulted during the planning stages of interventions through focus groups [10], this engagement rarely continues during implementation or evaluations stages of the research [4, 5]. Parents provide an important “lived experience” perspective that lends well to understanding both the family and participant experience firsthand; iKT approaches provide space to reframe and refresh what is traditionally considered “expertise” [11]. It is important to incorporate this experience throughout the process; opinions and experiences may change longitudinally and thus the feedback provided during early focus groups may not be appropriate or relevant later. Finally, when thinking about implementation, iKT approaches contrast with the top-down approaches to knowledge dissemination that are commonly used in child obesity prevention interventions (i.e., disseminating information through group classes, handouts, or emails). These top-down approaches do not ensure that information is relevant and interpreted or applied as intended [9], which can lead to the development of inappropriate and ineffective interventions.

iKT approaches have received increasing attention and credibility in healthcare over the past decade [12]. A recent review by Gagliardi and colleagues (2016) noted that while many studies report iKT successes, the specific iKT strategies that achieve beneficial outcomes remain largely unknown because few studies describe the logic or theory underlying the iKT strategy, provide a clear description of how and when knowledge users are involved, or provide a clear description of the evaluation process [13].

Although involving parents in childhood obesity prevention and intervention research is not a new idea, there are few examples in which parents are engaged throughout the entire research process, from initial needs assessment focus groups, through to the end-of-grant dissemination of findings. Two US-based obesity prevention interventions have engaged parents throughout the process via participant advisory groups [4, 14, 15]. The first, described by Jurkowski and colleagues (2013) included an advisory group made up of study participants and community members (90% female; number of fathers was not reported) [4]. The second, developed by Berge and colleagues (2016), describes a ‘citizen action group’ made up of parent community members (*n* = 9; 3 fathers, 6 mothers) and university researchers (*n* = 3) [14]. While both studies reported the impact of their respective interventions, detailed evaluations of the advisory groups themselves were not included [4, 14, 15].

The current builds on this research by describing the implementation and evaluation of a parent-only advisory council designed to engage participants in the implementation and evaluation of a longitudinal, family-based obesity prevention intervention.

The GFHS Family Advisory Council allows our study team to gain insights from participants regarding best approaches for recruitment, perceptions of and suggestions for the assessment and intervention protocols and suggestions for knowledge dissemination of study findings (end-of-grant KT), as well as to increase participant/family engagement. By providing a clear description of the theoretical basis, as well as how and when knowledge users are involved, and the evaluation used to assess the feasibility and impact of their involvement, this study will inform best practices for engaging participants in family-based research.

## Methods

### Research setting: the Guelph family health study (GFHS)

The GFHS is a longitudinal cohort study aimed at identifying early life risk factors for obesity and chronic disease and testing the long-term effectiveness of family-based interventions to promote healthy weight-related behaviours among young children (18 months – 5 years) and their families. Briefly, the GFHS intervention is conducted in the home and health educators trained in motivational interviewing visit families four times throughout the 6-month intervention period to help families create routines and set goals for healthy behaviours including sleep, physical activity, family meals, dietary intake and screen time. Following a formative assessment with key stakeholders in the community, including family health teams and public health, as well as focus groups with parents of young children (*n* = 28) [16], we conducted a year-long pilot study with 44 families (79 parents; 44 mothers and 35 fathers) to test the feasibility of our protocols. Results from this pilot study are published elsewhere [17]. The long-term goal of the study is to recruit 3000 families with young children from the Guelph area over the next 20 years.

### The GFHS family advisory council

The GFHS Family Advisory Council was initiated in August 2015 and meeting attendance rates have been 78% across the 7 meetings held from 2015 to 2017. Initiation of the Family Advisory Council included a specific aim to engage fathers in the advisory council. Despite the strong evidence of the important role fathers play in the development of their children’s health behaviours, including dietary intake [18, 19], physical activity [19, 20] and overall weight status [21], lack of engagement of fathers in obesity interventions has been identified as a key gap in childhood obesity prevention efforts [22]. Two invitation emails describing the Family Advisory Council and the duties required were sent to all parents who participated in our pilot (*n* = 79). The second invitation email was written to target fathers specifically. The Council meets formally three times per calendar year and provides feedback via email between meetings on time-sensitive matters. The Council is facilitated by a graduate student who serves as a liaison between the Principal Investigators (PIs) and the council to reduce any bias that may result from the presence of the PIs at the meetings. Meetings last an average of 1.5 h and are structured around an agenda that is co-constructed between the study team and the Council members. One week prior to meetings, the facilitator solicits agenda items from both the study team and Council members; Council members have a second opportunity to add items to the agenda during meetings. Meeting minutes are sent out to the Council members following each meeting to allow for additions and corrections, before being shared with the PIs and other study team members. Childcare, refreshments and free parking are available at each meeting. Parents receive an annual $50 grocery gift card honorarium for participating on the Family Advisory Council.

### Theoretical Foundation

**Fig. 1:**
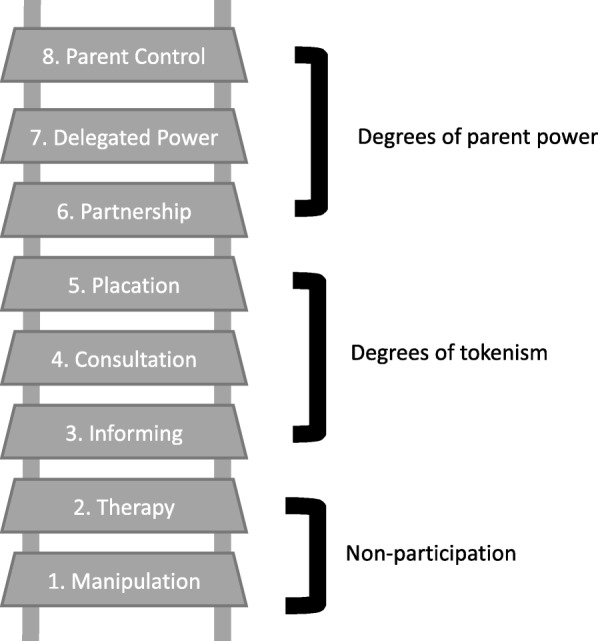
Ladder of Parent Participation. Modified from the Ladder of Citizen Participation [4, 23]

A limitation of existing iKT approaches is that few are based on theory or include a description of the theoretical foundation on which they are established [13]. Building off of previous research using iKT strategies to engage parents in child obesity prevention [4, 14], the GFHS Family Advisory Council was designed to achieve the highest levels of parent engagement on the Ladder of Parent Participation (See Fig. 1) [4, 23].

The Ladder of Parent Participation is based on the Ladder of Citizen Participation [23] and was created to help operationalize levels of parent involvement by describing who has power when important decisions are being made. The ladder has 8 rungs that represent increasingly higher levels of parent participation and engagement [23]. Briefly, rungs 1 and 2, *Manipulation* and *Therapy*, are non-participatory as the aim is to strictly cure or educate participants without consultation on the approach or messages disseminated [23]. While, *Informing* (rung 3), is an important step in all research processes, this is where engagement stops; the information flows one-way with no opportunity for feedback [23]. At rung 4, *Consultation*, parents are given a voice, usually for a brief length of time, but lack the power to ensure that their views are heeded by the research team [23]. Similarly, *Placation* (rung 5) is a higher level of tokenism, where parents are involved but have no decision-making power [23]. At this rung, parents may be consulted or hand-picked to be on an advisory committee, but the research team reserves the right to judge the legitimacy or feasibility of suggestions. Further up the ladder, power is redistributed. At rung 6, *Partnership*, planning and decision making responsibilities are shared (i.e. through joint committees made up of both parents and researchers, such as the advisory council described by Berge and colleagues [14] [23]. At rung 7, *Delegated Power*, parents hold the majority of the seats on committees with decision making power, which allows parents the assurance that the program or study is accountable to their ideas [23]. Finally, at rung 8, *Parent Control*, parents hold all the decision-making seats or have full managerial power (i.e. full planning and management of a program) [23].

Parents are often engaged between rungs 3 and 5, where they provide input and are notified of the study process, often on a one-time basis during formative planning stages, but do not have decision making power [4]. Further, parents are rarely involved at any rung on the Ladder of Parent Participation throughout the *entire* research process. Our goal was to achieve to parent engagement between rungs 6–8 throughout the entire process. While parents were informed and consulted during the early stages of the study design, the creation of our Family Advisory Council allows for a two-way channel of information sharing and partnership (rung 6). Parents co-create agendas with the facilitator and research team, allowing the Council to have power in the direction and topics discussed at each meeting (rung 7). Since the Family Advisory Council meets regularly, the research team is held accountable to the ideas presented by Council members. Further, the Family Advisory Council is made up solely of parent participants, ensuring that parent ideas are heard and not lost in the volume of other stakeholder groups (rung 8). To achieve and maintain the highest rungs of parent involvement, Council members are involved in as many study activities outside of the Family Advisory Council as possible. For example, Council members have presented on behalf of the GFHS at conferences, attended recruitment events and distributed recruitment flyers in the community, written letters of support for funding applications and participated as co-authors on grant applications (rungs 7–8).

### Evaluating the impact of the GFHS family advisory council

To assess the successes and impact of our Family Advisory Council, we conducted a process evaluation using an online survey in August 2015. The survey asked Council members whether the range and depth of discussion met their expectations and if the timing and structure of the meetings were appropriate. Attendance was also kept for each meeting.

To get a more in-depth understanding of why members joined the Council, what they liked and disliked about the Council and the impact they feel the Council has had on the GFHS, we also conducted a focus group in October 2016, 1 year following the initiation of the Council. The focus group was facilitated by a trained research assistant who was unknown to the Council members, using a semi-structured interview guide (See Table 1). The research assistant used prompts to encourage all participants to share their thoughts for each question [24]. The focus group was audio-recorded and transcribed verbatim. Qualitative analysis software (NVivo) was used for all coding procedures and we used directed content analytic methods [25, 26]. An a priori coding scheme based on the interview guide was employed; the key foci were: main factors that keep them engaged in the Council, personal benefits by participating the Council, Council’s benefit for and impact on the study, and areas for improvement for the Council. Two analysts independently read and coded the transcript line by line. Average agreement (%) between the two coders was calculated by the software. There was a high level of agreement between the two coders (93%). All discrepancies were discussed and resolved between the coders prior to analysis. Members of the Family Advisory Council reviewed the results and confirmed that the interpretations reflected their experiences. Informed consent was provided by all Family Advisory Council members who participated in the evaluation and ethics approval for the evaluation was provided by the University of Guelph Research Ethics Board (REB#17–03-015).

**Table 1:** Semi-structured interview guide used for Family Advisory Council Focus Group

1. You are busy parents of young children. What keeps you coming back to the GFHS Family Council meetings? 2. What do you feel you get out of attending the GFHS Family Council Meetings personally? 3. How do you think the GFHS Family Council adds to the study? What would be different about the study if it did not have a Family Council? 4. What changes in study protocol or engagement strategies have you seen the Family Council playing a role in? or: In what ways has the Family Council influenced how the GFHS has been implemented? 5. How can we improve the Family Council model? What, if any, changes would you like to see made to the way the Family Council is run?	

## Results

Seven parents (all mothers) responded to our first Council recruitment email. Following a targeted request for father participation in the Council, we recruited 7 more participants, including 4 fathers. In total, 14 parents (10 mothers, 4 fathers; 64% White) agreed to participate in the Family Advisory Council. The demographics of our Family Advisory Council reflect the pilot phase of the GFHS [17].

All 14 Council members completed the online survey. Overall, Council members expressed a very high level of satisfaction with the Council (results provided in Table 2). When asked why they were interested in joining the Family Advisory Council, parents identified that they were interested in being more involved in our novel research study, they wanted to help other families and that they wanted to give back to a study that they felt was helping their family lead a healthier life. Fathers seemed to be particularly appreciative of being included in the Council, as one father stated, “There was a specific call out for more dads and I’m very interested in this study overall, [so wanted to participate].”

**Table 2:** Survey Evaluation of the GFHS Family Advisory Council (*n* = 14)

Method of Evaluation	Result
Family Advisory Council Process Survey Evaluation	• 100% of Family Advisory Council members felt the topics discussed were what they expected and felt the depth of discussion was appropriate. • 85% of Family Advisory Council members were very comfortable sharing their opinions in a group setting; remaining 15% were comfortable. • 100% of Family Advisory Council members were very satisfied with the organization of the meetings.
Outcome Evaluation	• 92% of Family Advisory Council members responded to emails requesting quick study feedback (i.e. recruitment ideas) • 78% average attendance at Family Advisory Council meetings. Members who have been unable to attend meetings have provided feedback either prior to the meeting via email or following the meeting via additions to the minutes.

### Family advisory council focus group

Nine members of the Family Advisory Council participated in the focus group (7 mothers; 2 fathers). Results are presented below in four main themes derived from the coding scheme: main factors that keep participants engaged in the Council, personal benefits by participating on the Council, Council’s benefit for and impact on the study, and areas for improvement for the Council.

### Factors for engagement

Members were asked what kept them coming back to Council meetings as busy parents and they identified three main factors that keep them engaged: convenience, having facilitators who value their time and having a greater connection to the study.

With regards to convenience, parents like the timing of the meetings and that food, parking and childcare are provided. As one parent said, “…it’s easy to come to, there’s childcare providers, there’s free parking, it’s at time that works, its only three times a year. It’s a very easy thing to do really, it’s not um, for me at least it’s no problem at all, its enjoyable”. Another parent stated, “And like we’re meeting at a time, you know, where we all need to eat so they’ve got food provided for us. You know, just like thoughtful things considering we’re doing a health study…”.

Members identified that they continued to participate because they feel the facilitators were welcoming and valued their input. One parent stated, “Yeah, we really have an input. And it’s wonderful that they value that”. Another said:

“I think that the facilitators are very welcoming and open, and I think that really helps. They obviously are very passionate about what they’re doing, and I think you know, I’ve felt that through the whole time […] I think that you know if they weren’t like that, I’d probably be like pfft whatever, like you’re not gonna wanna participate as much.”

With the longitudinal nature of the study, members stated that the Council was a way for them to stay closer to the study. For example, one mother staid,

“Because it’s such a long time-frame too it’s nice to… stay connected in that same way. A little bit more so than just coming in once a year to put on your activity monitors and all that stuff…”.

An interest in being closer to the results of the study was also common among parents, with one parent talking about being disappointed in a previous study experience:

“I did a study at [university name] when [daughter] was little and I always wondered what the results for that study were. It was a really interesting study. […] we went along and did this little thing and then they gave me my gift card and I left and never heard another word about it. And I often like actually google and see…I wouldn’t even know what to google though. I have nothing to show for it. And I’d be really interested in the results, and here, we’re involved, and we do know.”

### Personal benefits

Beyond the Family Advisory Council being convenient and a way for members to feel connected to the study, the experience has also provided many of our members with personal benefits. Members mentioned that the experience made them feel valued as a person and allowed them an opportunity for personal or professional development. As one parent stated, “Like it can be a really great thing to add value to your own credentials”. Another parent said:

“It gives me a sense of pride and a feeling of importance that, yeah, my opinion is valued. I’m a blue-collar worker now. I used to be an accountant but now I’m a school bus driver, so I’m not necessarily looked at as being you know the CEO or anything, like my opinion isn’t always valued on some things. So, this, it feels like you know, it gives you that sense of worth and importance, that you’re making a difference and contributing.”

Members also talked about the Council as a time for them to connect with other adults. A single parent noted, “It’s nice to feel like an adult in participating. With young kids, often, I’m sure other people can relate, is that you kinda get into that mom role and it’s nice to step out and maybe be a bit more academic or a little more, you know, adult”.

### Benefit for and impact on the study

Members highlighted many benefits that they feel the Council added to the GFHS. Specifically, parents feel that the Council provides a fresh perspective to the research design and helps increase participant engagement. As one mother stated, “I think as lay people, so to speak, we have a different set of eyes than the researchers or developers will have so I think that helps as well. Maybe not just the participant perspective, but just as you know every day people”. A father noted that the input the Council provides helps make it easier for other families to participate: “I think it helps future families coming into the study, because if something’s worked better than others, then being able to change it a bit so that it’s easier as the project moves forward […] is really useful and beneficial”.

As an extension of feeling that their opinions are valued by the facilitators of the Council and the larger research team, members highlighted many examples of times when their feedback has had direct impact on the study design or methods. As one parent summarized:

“They asked us around that questionnaire and stuff a few umm meetings ago to try and help streamline some of their questionnaire, so we helped to simplify that. And the newsletters as well, we also commented on […] how often we wanted them, what sort of content we wanted to receive, and then I’m just thinking the wrist bands too. We’ve had input on the recipe book, and the blood testing stuff we mentioned”.

### Areas for improvement for the council

While members had many positive things to say about the Council, areas for improvement were highlighted, including connecting more easily as a group and planning for the future.

Parents were interested in ways to connect more easily as a group and with other parents participating in the study. Social media was highlighted as a potential avenue for this increased connection:

“You know I think I come back to social media because we use it all the time. Like Facebook has groups and people can post comments and people can reply to comments and you know there’s just that sort of an openness to things, not that there isn’t here, there very much is, but maybe create some more fluidity throughout the year”.

Members were thoughtful of the diversity of the Council and commented on the importance of ensuring that it is reflective of the larger study as it grows. As one member stated, “I mean, just looking around, like obviously it is great we have some dads here too…but you know maybe that’s one thing trying to you know make sure the Family Council diversity is reflective of the diversity we see as a whole…”. Beyond the Family Advisory Council itself, members also commented on the future of the GFHS and highlighted ways that they feel the study will need to adapt to stay relevant as it grows. For example, as the child participants age, study incentives may need to move from family incentives (grocery gift cards) to incentives that are also teen focused. Members also brought up the idea of having a similar council for the children in the study when they get older, “If this is going to be a 20-yearlong study then these kids are gonna be preteens, teens and I think it would be nice to involve them”. Another parent built on this idea:

“…like when they get to be teenagers, why can’t they have a group like this? That, they’re sitting down and they’re getting asked questions so not necessarily a survey but they’re sitting down as the council of initial children to say that yeah these are the changes that I’ve felt and how its impacted my life or not”.

### Impact of the family advisory council on GFHS engagement and retention

Input from the Family Advisory Council has informed the implementation of the GFHS. Table 3 describes examples of study initiatives and changes that have resulted from Council feedback. It is likely that these efforts have facilitated the very high retention rates during the study’s pilot year; 44 families completed baseline assessment and were randomized to the study and 42 families completed the 6-month follow-up assessment (95% retention rate). Furthermore, Family Advisory Council input on recruitment ideas allowed us to implement very successful recruitment strategies for the next phase of the GFHS; we recruited 40 families within 5 weeks into the second phase.

**Table 3:** Examples of GFHS Family Advisory Council feedback and resulting study changes

Study issue discussed	Changes implemented based on council feedback
• Ideas for how to increase outreach and engagement with families participating in the Guelph Family Health Study.	• Provide families with mailed birthday cards and holiday-themed emails. • Send health reports to families with information from 6 and 18-month study Health Assessment visits (height and weight for children; blood pressure, heart rate, % fat mass, % fat free mass for adults). • Six families provided written testimonials, family pictures and recorded videos to highlight their study experiences on our social media outlets (Facebook, Twitter and study website). • Facebook posts created to maintain the GFHS’s online presence and activity within the community. Parents indicated that along with updates about the study, the GFHS is a trusted source of information for them, so posts linking them to healthy recipes and trusted health articles would be welcome. • A GFHS blog will be created in Fall 2017 on our website with monthly posts related to family health as well as guest posts from study participants about their experiences in the study.
• To help busy families have more family meals and improve their dietary intake, parents requested recipe ideas for quick, easy and healthful meals.	• The GFHS created a crowd funding initiative in Dec. 2015 to develop three seasonal recipe books with easy, kid-friendly, quick and healthy meal ideas. These online books were distributed free to participating families (following feedback from the Family Advisory Council on format) and can be accessed free here: https://guelphfamilyhealthstudy.com/cookbooks/
• Parents reviewed the GFHS consent form and the University of Guelph Research Ethics Board (REB) to provide a participant perspective.	• A report was created for the University of Guelph REB to assist with the creation of new university-wide online consent forms.
• Parents reviewed and requested more detailed information be included in the intervention emails. Parents also provided insight towards the moving the study’s health behaviour messages from email to text delivery.	• Changed intervention messages to include more multi-seasonal content as well as links to access more detailed information for interested parents. • Parents indicated that texts provide the opportunity for in-the-moment reminders for healthy behaviours and are more convenient for busy families. The study team is working on changing to a text-based format for the delivery of the bi-weekly (intervention) and monthly (control) health behaviour messages.
• Parents reviewed and provided feedback on new study questionnaires to assess food skills and food purchasing habits of families with young children. Parents pilot-tested the survey in Winter 2016.	• Questionnaires assessing food skills and food purchasing habits were updated to include Council suggestions such as including questions about using technology (i.e., apps) to assist with food preparation.
• Parents provided suggestions on how to make children’s accelerometers easier to wear.	• We are now sending accelerometers with extra wrist bands to use as replacements if bands get wet. We have also purchased bands with varying colours to increase the “kid-friendliness”. • The study has also moved to having the children wear the accelerometers on the hip only (vs. hip and wrist) as children seem to prefer the hip bands.
• Parents provided their thoughts towards sources of funding for the study (i.e. research grants vs. industry funding)	• Based on parent feedback, the GFHS will not explore sources of funding from industry. Parents expressed concern with the bias that such sources may have on the study or the perceptions that such funding may put on the study within the community.

## Discussion

The goal of the Guelph Family Health Study Family Advisory Council is to bridge the gap between science and practice while addressing the common challenges of longitudinal research including continued retention and engagement. While previous obesity prevention interventions have involved parents between the third and fifth rungs of the Ladder of Participation [10], this is the first known Canadian study to involve parents throughout the entire research process at the highest level rungs of participation [4]. Engaging participants throughout the entire study process is important because participants are the experts of their needs and understand the realities of the environments where the research will be implemented and disseminated; realities that researchers may not be aware of and that are ever-changing [8].

This was also the first family-based obesity prevention intervention to evaluate the feasibility of the Council, as well as the impact of the Council on the research study. Overall, we found the Council process to be feasible with high attendance rates over 2 years. While previous research has reported that involving parent participants who are typically busy with multiple responsibilities is a barrier to successful iKT research [13], our attendance findings do not reflect this. Our participants, who are parents of young children, including single parents and those working full-time, identified that convenience was an important facilitator to their participation. By providing food, parking and daycare and by working with the participants to schedule the meeting times, we helped ensure the meetings were accessible for our participating parents. Our results highlight the importance of considering the time and contextual constraints of participants in iKT efforts to ensure participation throughout the research process.

In addition to convenience, we found that the value the facilitators’ place on Council members’ time and input is a key factor for engagement. These findings reflect the enablers to iKT research previous described in the literature [13], including having strong leadership who have formal training in iKT and a commitment to the process of shared decision making. Having facilitators who value and reflect the importance of a shared research partnership with participants not only ensures that the iKT strategy is fully implemented and utilized, but enhances participant buy-in [13]. Further, building rapport between facilitators and parents is key to ensuring a transparent relationship and a space for parents to feel comfortable sharing their opinions and experiences [13]. Strong facilitators also make participants feel connected to the study; members indicated that participating in the Council made them feel closer to the study and the data being produced. This connection is paramount for the long-term engagement that is required to sustain longitudinal iKT research. Careful selection and training of group facilitators is important to iKT efforts to ensure participant buy-in and sustainable engagement. To maintain a neutral position, to be in-service of the spirit of true collaboration and to allow members to express their opinions freely, facilitators should not be members of the research team.

Members also identified that the Council provides them with an opportunity to connect with other adults and provides a unique avenue for personal and professional development. This opportunity to be involved in multiple and varied opportunities within the study has been found to be an enabler of successful iKT strategies in previous research [4, 13]. Providing opportunities for parents to connect and identifying various ways to engage them in the research, (i.e., direct engagement through recruiting other families or being involved in applying for funding for future projects), may be an important approach for sustaining Council members’ participation in a Family Advisory Council.

Council members acknowledged that seeing the impact the Council has had on the GFHS is a key benefit of participating on the Council. Previous iKT research has also identified the importance of having participants influence the policy making and service delivery of the study [13] due to the unique perspective they provide. Reporting back to Council members to demonstrate how their input is impacting the delivery of the research study is key to developing a trusting and transparent partnership. Previous research suggests that ensuring this high level of impact may also be important to the sustainability of iKT initiatives such as a parent council. Jurkowski and colleagues [4] reported high levels of parent participation during the initial phases of their community advisory board, but attendance levels dropped as the group had fewer decisions to make. Future efforts should focus on sustained involvement in decision making to help increase retention and participant engagement in iKT interventions, such as the Guelph Family Health Study.

### Strengths and limitations

Results of this research should be considered in light of a number of limitations. First, while the parents participating in our Council are reflective of the demographics in the GHFS, the findings of our evaluation may not be generalizable to other research settings outside of the home. Second, our evaluation was conducted with members of the Family Advisory Council and does not include the perspectives of the larger GFHS parent population. Future research should also assess how the Family Advisory Council has impacted the study from the perspective of the family participants. Third, our iKT efforts were not examined with respect to study outcomes to assess whether the increased engagement created by the Family Advisory Council translated into meaningful weight change outcomes for the GFHS pilot. Future research should assess the impact of sustaining our iKT efforts on the outcomes of future waves of the GFHS.

## Conclusions

In sum, our findings highlight the importance of engaging families at higher rungs on the Ladder of Parent Engagement in order to support successful iKT research [13]. Briefly, we found that the GFHS Family Advisory Council is a feasible and successful way to increase parent engagement in a longitudinal childhood obesity prevention intervention; members of the council reported appreciating the opportunity to be closer to the study, the feeling of being valued and the opportunity for professional development. Major facilitators of the Council’s success included convenience, strong facilitation and the members’ feeling that their input is valued and has had an impact on the study. While childhood obesity prevention interventions often do not see significant changes in weight outcomes [6, 7] and longitudinal research designs are limited by attrition [27], results suggest that incorporating an iKT approach into the research design holds promising solutions to both. Engaging mothers *and* fathers in all stages of the research process, from formative work through to end-of-grant KT, allows researchers to ensure that the intervention messages and outcomes are relevant and meaningful to the participants and that the study protocols are feasible and acceptable to families; all of which can lead to improved behaviour change and participant retention. The GFHS Family Advisory Council is a novel example of how an iKT approach can benefit longitudinal family-based cohort studies.

## Abbreviation

BMI: Body mass index
GFHS: Guelph family health study
iKT: Integrated knowledge translation
